# Current Knowledge and Computational Techniques for Grapevine Meta-Omics Analysis

**DOI:** 10.3389/fpls.2017.02241

**Published:** 2018-01-09

**Authors:** Salvatore Alaimo, Gioacchino P. Marceca, Rosalba Giugno, Alfredo Ferro, Alfredo Pulvirenti

**Affiliations:** ^1^Bioinformatics Unit, Department of Clinical and Experimental Medicine, University of Catania, Catania, Italy; ^2^Department of Computer Science, University of Verona, Verona, Italy

**Keywords:** *Vitis vinifera*, microbiome, metagenomics, metatranscriptomics, bioinformatic tools and databases

## Abstract

Growing grapevine (*Vitis vinifera*) is a key contribution to the economy of many countries. Tools provided by genomics and bioinformatics did help researchers in obtaining biological knowledge about the different cultivars. Several genetic markers for common diseases were identified. Recently, the impact of microbiome has been proved to be of fundamental importance both in humans and in plants for its ability to confer protection or induce diseases. In this review we report current knowledge about grapevine microbiome, together with a description of the available computational methodologies for meta-omics analysis.

## 1. Introduction

*Vitis vinifera* is one of the most important plant in modern agriculture. Its economic and cultural impact is undeniable (Mullins et al., 1992; Pulvirenti et al., 2015). Almost 8 million hectares of vineyards (Vivier and Pretorius, 2000) together with 5, 000 estimated cultivars (Jackson, 1994) make grapevine economic contribution to wine-producing countries very significant. Improving wine quality and increasing grapevine pathogens and environmental stress resistance is crucial for wine industry (Vivier and Pretorius, 2002).

In 2007, the French-Italian Public Consortium for Grapevine Genome Characterization sequenced the first genome of *V. vinifera* Jaillon et al. (2007). Grapes genome has driven a multitude of studies on the genetics of grapes (Tomkins et al., 2001; Adam-Blondon et al., 2005, 2011; Lamoureux et al., 2006). Research attention has been also focused on grapevine transcriptome analysis. Gene expressions and trascriptional profiling data analysis have shed light on several *V. vinifera* biological processes. In particular the following biological functions have been considered: (i) ripening and maturation (Fortes et al., 2011; Guillaumie et al., 2011; Fasoli et al., 2012; Lijavetzky et al., 2012); (ii) dormancy transitioning (Sreekantan et al., 2010); (iii) and resistance to pathogens and environmental conditions (Grimplet et al., 2009; Polesani et al., 2010; Tillett et al., 2011; Pulvirenti et al., 2015). Nowadays, an increasing amount of knowledge proves the importance of beneficial plant-associated microbes in plant health, growth, nutrition and stress resistance, as well as in increasing crop quality thanks to their biostimulant properties (Rouphael et al., 2015; Pieterse et al., 2016). From a functional point of view, plant microbiome is comparable to gut microbiome in mammals, and it has been defined as the plant's second genome. In this perspective, plant fitness (a quantitative description of survival and reproductive success of a plant in a given environment) is the point of convergence of two components: the plant itself and its associated microbiota, which collectively form a holobiont (Vandenkoornhuyse et al., 2015). Indeed, it has been shown that grapevine growth and survival are significantly impacted by its microflora. This latter has been identified as a key factor influencing not only plant fitness, but also many vine traits, positively contributing viticulture economy. Many viticulturists are now persuaded that wine organoleptic properties are partly due to microbes influence.

One of the most accepted principle in this field concerns the beneficial effects of *mycorrhizal symbiosis*. Indeed, grapevine growth is remarkably dependent on mycorrhizae, since this plant has low-density roots and few root hairs. Mycorrhizal fungi improve water use efficiency, soil nutrient uptake, and biomass production in grapevine. Furthermore, arbuscular mycorrhizal communities composition in vineyards is largely influenced by surrounding vegetation (Holland et al., 2014). According to the latest studies, based principally on culture-independent methods, the eukaryotic microbiome of *V. vinifera* is characterized by fungi belonging to early diverging fungal lineages, *Ascomycota* and *Basidiomycota*. On the other hand, prokaryotic microbiome is prevalently composed by *Proteobacteria*, followed by *Firmicutes, Actinobacteria* and *Bacteroidetes*. At species level, these microorganisms, either *epiphytes* or *endophytes*, are not uniformly distributed along the vine. Belowground microbial communities significantly differ from aboveground ones. More precisely, the level of biodiversity decreases from belowground to aboveground. Furthermore, aboveground microbiota is subject to temporal variation during grapevine vegetative cycle and agricultural practices like integrated pest management (Pancher et al., 2012; Martins et al., 2013; Campisano et al., 2014; Pinto et al., 2014; Zarraonaindia et al., 2015). In healthy conditions, within the aboveground fungal microorganisms, the *Aureobasidium* genus results to be predominant and ubiquitous all over vine's organs, including leaves and barks. In this group, *A. pullulans* is by far the most abundant fungal species. *Cryptococcus* spp. and *Rhodotorula* spp. are frequently found on leaves and grapes with a high relative abundance, while *Candida* spp. and *Pichia* spp. are more present on grapes than other parts of the plant. All together, these microorganisms are capable of exerting antibacterial activity, inhibiting spore germination and mold growth, resulting in a protector effect on vine and grapes (Raspor et al., 2010; Mousa and Raizada, 2013). Similarly, bacterial species of the genera *Pseudomonas* and *Bacillus* are widely spread on flowers, leaves and grapes. Genera *Burkholderia, Sphingomonas, Serratia* and *Streptococcus* are more present on leaves and grapes. Finally, *Erwinia* spp. are dominant in flowers. These bacterial genera are among the most abundant in grapevine and some of them are well established bacterial and yeast antagonists (De Vleesschauwer and Höfte, 2003; Trotel-Aziz et al., 2008; Elshafie et al., 2012). Besides beneficial microbes, metagenomic analysis also revealed the presence of phytopathogen microorganism living on grapevines, albeit normally with low abundance, like fungal species of the genera *Phomopsis, Cryptovalsa*, and *Botryotinia* (Pinto et al., 2014). Keeping a balance between beneficial microorganisms, in terms of abundance and richness, is of crucial importance for grapevine health and biocontrol of pathogens.

Grapevine microbiota is influenced by several factors, including pedoclimatic and biogeographic conditions. The concept of a region-specific microbiota gives strength to the concept of “terroir,” on which viticulturists often heavily rely. Not all regions and vineyards are microbiologically unique, but evidences prove the existence of patterns at least on a large scale for microbial communities in grapes and musts. These correlate with climate, soil type and crop management (Bokulich et al., 2016). On the other hand, it is important to stress that studies on the biogeographic structural characterization of the grapevine microbiota have been mainly carried on grape samples. Furthermore, there is a lack of information regarding the role of soil microbiome in defining the terroir at a local scale, as well as the influence exerted by grapevine roots on soil microorganisms and vice versa. A recent study suggests that soil microbiome could represent a potential reservoir, since about 40% of bacterial OTUs obtained from grape, leaf and flower samples are also present in root samples. Moreover, about 48% of prokaryotic OTUs from three different types of belowground samples overlap (Zarraonaindia et al., 2015). Rolli et al. reported that plant growth promoting bacteria (PGPB) of different geographical origins and different crop plants rapidly colonizes the root system of grapevine. This allows the growth of grapevine under different field conditions. However, authors found contrasting results in the literature concerning the effect of PGPB on different plants. This clearly suggests that a more in dept analysis is needed (Rolli et al., 2017).

## 2. Computational methods for microbiome analysis

Given the recent interest in meta-omics sciences, many computational methodologies are rising to allow the interpretation of the large amount of data produced by high-throughput techniques. The primary purpose of these studies has been the identification of microorganisms present in a environmental sample, determining their activity and interaction with host plant.

Metagenomic methodologies, developed to establish microbiome composition, are divided in two classes: DNA Metabarcoding techniques and Genome Relative Abundance (GRA) estimation techniques.

DNA Metabarcoding aims at identifying a set of *operational taxonomic units* (OTUs) present in a single environmental sample. However, this method requires specific algorithmic techniques capable of handling large amounts of data. QIIME (Quantitative Insights into Microbial Ecology: Caporaso et al., 2010) is a suite of tools combined to define standard pipelines for metabarcoding analysis. It provides a set of analysis and prediction algorithms, along with graphical reports, allowing a simplified analysis of the results. OBITools (Boyer et al., 2016) is another tool enabling analysis from raw sequencing data up to taxon assignment. PRINSEQ (Schmieder and Edwards, 2011) offers functionality similar to QIIME and OBITools through a web interface. Several other tools are available: UPARSE (Edgar, 2013) aims to detect *de novo* OTUs from NGS reads achieving high accuracy in biological sequence recovery, and improving richness estimate; MOTHUR (Schloss et al., 2009) is a comprehensive software package, which analyzes community sequencing data; DADA2 (Callahan et al., 2016) is a model-based approach to correct amplicon errors without constructing OTUs. See Table 1 for an overview of described methods.

**Table 1:** Brief description of metabarcoding: tools advantages and disadvantages.

**Tool**	**Bioinformatics tools for metabarcoding**		
	**Brief description**	**Advantages**	**Disadvantages**
QIIME	Pipeline for performing microbiome analysis by exploiting a set of integrated scripts for analyzing raw microbial DNA samples, including taxonomic classification using marker genes.	Allows flexible multi-script pipelines to be constructed. Allows wide statistical analysis with advanced graphical visualizations. Provides compute resources for free.	Command line interface. Installation on local machine may be difficult for non-experts. Not multi-platform.
OBITool	Set of programs specifically designed for analyzing NGS data in a DNA metabarcoding context, designed to target microbial communities from various ecological contexts.	Relies mainly on filtering and sorting algorithms, allowing users to set up flexible data analysis pipelines. It takes into account taxonomic annotations, allowing sorting and filtering of sequence records based on the taxonomy.	Command line interface. Installation on local machine may be difficult for non-experts. Not multi-platform.
PRINSEQ	Projected to trim adapter sequences and low quality ends and to remove the reads containing ambiguous nucleotides and duplicate reads from the sequencing data output, accelerating read data analysis.	User-friendly. Generates complete statistics of data-seq for parameters like sequence length, GC content, quality score and replicates. Capable of treating both single and paired-end reads. Exploitable also for metagenomics and metatranscriptomics data.	Window size needs to be defined by users for the initial trimming step. Limited to pre-processing.
MOTHUR	Principally designed to target the microbial ecology community, it provides an extensible package with functionality accessible through a domain-specific language. It incorporates algorithms from previous tools plus additional features.	Single program for complete analysis with basic visualizations.	Custom command line interface. Incomplete usage of software engineering techniques. Not multi-platform.
DADA2	R package implementing the full amplicon workflow, from filtering to merging of paired-end reads.	Uses a statistical model of amplicon errors to infer sequence variance instead of construct OTUs. Very high accuracy.	Command line interface.

The main shortcoming of metabarcoding is the classification of OTUs using an existing reference, and the preparation of specific sequencing libraries (Somervuo et al., 2016). Recently, techniques have been developed to detect microbial composition directly from shotgun sequencing. These approaches can be divided into two categories: compositional-based and alignment-based (Xia et al., 2011). In compositional-based approaches, *k*-mer frequency measurements are used to classify metagenomic reads. Methods such as TETRA (Teeling et al., 2004), CompostBin (Chatterji et al., 2008) and TACOA (Diaz et al., 2009) organize sequences in clusters (*k*-mer frequency is used to build a feature vector to compute distances between reads). Next, they assign an unique taxon to each cluster through a set of references computed on known genomes. However, none of these approaches is able to estimate Genomes Relative Abundance (GRAs) for microbial communities. AbundanceBin (Wu and Ye, 2011) uses the content of *k*-mers in the reads to estimate abundance of the genomes. The main assumption in this process is that reads are sampled from genomes following a Poisson distribution. However, detection efficiency decreases when a uniform distribution of species is present in a sample.

Unlike compositional-based algorithms, alignment-based methods use tools such as BLAST to find similarity to a reference species database, while estimating the relative abundance of each genome. MEGAN (Huson et al., 2007) uses BLAST to assign a species to each read, tracing the lowest common ancestor for those with multiple assignments. Then, it estimates the relative abundance using reads distribution normalized by taking into account ambiguous ones. GRAMMy (Genome Relative Abundance using Mixture Models: Xia et al., 2011) uses BLAST to perform an initial assignment of the species to each read. Next, it uses Expectation Maximization (EM) technique to establish probability assignment of the reads, modeling ambiguities, and accurately identifying the relative quantities of each species. See Table 1 for an overview of described methods.

Although the metagenomic approach can estimate a profile of a sample microbial community, it only allows comparative studies in different conditions, without providing insight on their actual activity (Simon and Daniel, 2011). A complementary view is given by metatranscriptomics. It gives details on the expression profiles and regulation mechanisms in the identified microorganisms (See Table 3 for an overview of all methods).

This produces details concerning progress and intensity of biological and metabolic processes, elucidating the means by which a microbial community interacts with its host. Several tools can analyze RNA-seq data to extract information about microbial transcriptome. In Leimena et al. (2013), authors propose a comprehensive platform for metatranscriptomics analysis. It allows removal of rRNA sequences, prediction of taxonomic origin and assignment of a function to mRNAs in a sample. HUMAnN2 (Abubucker et al., 2012) can detect the presence, absence, and abundance of microbial pathways through sequencing data. The purpose of the suite is describing the metabolic potential of a microbial community and its members, establishing a functional profile. MetaTrans (Martinez et al., 2016) is an open-source pipeline, which analyzes the structure and function of an active microbial community in a sample. It was developed to analyze large amounts of data produced by sequencing leveraging parallel computing techniques. COMAN (Ni et al., 2016) is a tool for determining the metatranscriptome through an easy-to-use web interface. The primary purpose of the platform is providing tools for quality control, metatranscripts counting, and several statistical analyzes. It can be used in the absence of sufficient computational resources and without any programming expertise, since it is based on a web interface. See Table 2 for an overview of described methods.

**Table 2:** Brief description of GRA estimation tools: advantages and disadvantages.

**Tool**	**Bioinformatics tools for Genomes Relative Abundance (GRA) estimation**		
	**Brief description**	**Advantages**	**Disadvantages**
TETRA	Pioneering classifier that uses tetranucleotide-derived z-score correlations to taxonomically classify genomic fragments. Compositional-based.	Provides statistical analysis of tetranucleotide usage patterns in genomic fragments. It works either via a web-service or a stand-alone program.	Accuracy at genus level is reached using long reads (>1 kb). Tends to create multiple clusters for reads originating from highly abundant species when the sample contains multiple species with highly varying levels of abundance.
CompostBin	DNA compositional-based algorithm which adopts a weighted Principal Component Analysis (PCA)-based strategy. Compositional-based.	Reduces the dimensionality of compositional space. Bins raw sequence reads without need for assembly or training.	Accuracy at genus level is reached using long reads (>1 kb). Tends to create multiple clusters for reads originating from highly abundant species when the sample contains multiple species with highly varying levels of abundance.
TACOA	Multi-class taxonomic classifier combining the idea of the k-nearest neighbor with strategies from kernel-based learning. Compositional-based.	Easily installed and run on a desktop computer. Its reference set can be easily updated with newly sequenced genomes.	Accuracy at genus level is reached using long reads (>1 kb).
AbundanceBin	Binning tool, based on the l-tuple content of reads, developed on the assumption that reads are sampled from genomes following a Poisson distribution. Compositional-based.	Capable to return accurate results also when the sequence lengths are very short (~75 pb).	Binning efficiency decrease in case of samples which tend to have a uniform distribution of species.
MEGAN	Standalone computer program allowing large metagenomic data sets. It uses BLAST or other comparison tools to assign species to each read, and then employs the NCBI taxonomy. Alignment-based.	Allows large data sets to be dissected without the need for assembly or the targeting of specific phylogenetic markers. Provides statistical and graphical output. Computes quantitatively accuracy and specificity.	Uses bit-score of individual hits as the sole parameter for judging significance, thus affecting specificity and accuracy of taxonomic assignments in different scenarios.
GRAMMy	Probabilistic framework developed for GRA. It uses the Mixture Model theory.	Exploitable with mapping, alignment and composition-based tools. Possibility to handle very short reads obtaining accurate results.	Accuracy in estimated abundance decreases in case of closely related microbes whose genomic sequences are highly similar.

**Table 3:** Brief description of metatranscriptomics tools: advantages and disadvantages.

**Tool**	**Bioinformatics tools for metatranscriptomics**		
	**Brief description**	**Advantages**	**Disadvantages**
HUMAnN2	Pipeline for profiling the presence/absence and activity level of microbial pathways in a community.	Easy to install and extensive documentation and examples. Uses commonly available tools and databases.	Command line interface.
MetaTrans	Pipeline aiming to analyze structure and functions of active microbial communities using the power of multi-threading computers.	Its design facilitates the inclusion of third-party tools in each of its stages. Possibility to perform RNA-Seq analyses addressing both 16S rRNA taxonomy and gene expression.	Installation on local computer may be difficult for non-experts. Require proper local setup on a powerful computer.
COMAN	Web-based tool dedicated to automatically and comprehensively analyzing metatranscriptomic data.	Easy-to-use interface and extensive instructions for non-experts. Processes uploaded raw reads automatically to ultimately achieve functional assignments, which are then exploited to perform further analysis.	Web-based interface not suitable for big analysis.

An important limitation of the methods analyzed so far is the use of functional enrichment to establish microbial pathway activity. Recently, a new paradigm has emerged. Indeed, considering both gene expression and their interaction network can lead to more accurate results (Tarca et al., 2008; Alaimo et al., 2016, 2017). These tools have great potential for microbial activity analysis and quantifying microbial interactions with the host, through its pathways.

## 3. Manipulating grapevine microbiome: from *in silico* to the field

Traditionally, to overcome the low economic returns caused by Grapevine Trunk Diseases (GTDs), viticulturists treat plants with pesticides. Commercially available microbial inoculants are also available. Most of these inoculants includes individual bacterial or fungal strains, aiming to contrast grapevine pathogens without causing environmental pollution (McSpadden Gardener and Fravel, 2002). However, despite the availability of registered biocontrol products, recently published data report that their adoption in viticulture is still limited mainly due to the belief that they are less effective than traditional pesticides (Gramaje and Di Marco, 2015).

The growing understanding of microbial influence on plants is driving us toward an innovative sustainable viticulture where microbes will replace pesticides, improving grapevine traits. In this new scenario, culture-independent molecular techniques and *in silico* analysis are the new protagonists. Metagenomics, metabarcoding, metatranscriptomics and other molecular approaches applied to healthy, pesticides-treated, and disease-affected grapevines are revealing specific patterns of colonizing microorganisms. This will enable the development of prediction models based on structure and transcriptional profile of organ-specific vine-associated microbiome.

Some recent works focused on GTD-affected vines highlighted the importance of microbial communities influence. In field conditions, plant infectious diseases are rarely due to single host-pathogen interactions. They are the result of simultaneous biotic and abiotic stresses on plants, which induce a sequential colonization process of the host tissue (Travadon et al., 2016; Song et al., 2017). Beneficial microbial communities work as a barrier defending against plant pathogens. This reduces the potential of pathogens invasiveness, since a significant fraction of their niche overlaps (Wei et al., 2015).

In *Esca*-affected vines the fungal community structure undergoes a considerable change in comparison with healthy plants (Morales-Cruz et al., 2017). In apparently healthy vines, very low fungal counts were recorded. *Phaeomoniella chlamydospora* and *Phaeoacremonium minimum* (two Esca pathogens) appeared to be relatively more abundant than other taxa. The most variable fungal composition was reported in vines with wood symptoms but no foliar symptoms, with a generalized notable increase in abundance and activity of pathogenic fungal taxa. Furthermore, *P. chlamydospora* and *P. minimum* were reported to be highly predominant in wood together with *Diaporthe ampelina* (causal agents of *Botryosphaeria dieback*). No significant changes were reported for the bacterial community, since the nine most abundant species belonged to the genera *Bacillus* and *Pantoea*. However, a deeper analysis of the bacterial community is required, especially from a functional point of view (Bruez et al., 2015). For instance, the antagonistic activity of two microbial strains of *Bacillus pumilus* and *Paenibacillus* sp. against *P. chlamydospora* was recently tested *in vitro*. These two strains can synthesize volatile compounds with antifungal activity. Furthermore, *B. pumilus* inoculation confers a systemic resistance in grapevine (Haidar et al., 2016). Such experiments indicate that pathogen detection methods aiming to differentiate between the early and late stages of infection should be quantitative (Morales-Cruz et al., 2017). Therefore, the usage of metatranscriptomics tools, in combination with pathway analysis, of healthy and affected plants, might elucidate the functional relationships between microbial communities, leading to the discovery of novel interactions.

The fungal pathogen *Eutypa lata* is predominant in wood tissues of *Eutypa dieback* affected vines, followed by high abundances of *Diplodia seriata* and *Phaeomoniella chlamydospora* (Morales-Cruz et al., 2017). No information is available concerning the bacterial community changes with respect to this disease. Yet, it was shown that in crown gall affected grapevines, changes in bacterial community is site-specific since a shift in composition happened only in graft unions. *Agrobacterium vitis* is the infectious agent causing the disease (Faist et al., 2016). Authors recorded that the difference in microbial community composition was due to nine bacterial species. The most abundant ones were *A. vitis, Pseudomonas* sp. and *Enterobacteriaceae* sp. However, it was determined that the induction of this disease by *A. vitis* do not necessarily requires a core microbiome. Morales-Cruz et al. (2017) detected some pathogenic fungi also in asymptomatic samples, especially *P. chlamydospora* and *P. minimum* with a ratio of *1:200* in respect to GTD-affected samples. Therefore, a more in-depth metagenomic analysis is needed in order to elucidate the composition of the microbial community, with a greater effort on pathological strains.

Metatranscriptomic analysis and functional profiling also helps identifying biological processes linked to specific conditions. For example, Morales-Cruz et al. (2017) showed that most virulence-related expressed genes belonged to carbohydrate active enzymes and transporters, followed by genes related to secondary metabolism, cytochrome P450s and peroxidases. In addition, authors demonstrated that it is possible to distinguish the *Esca* pathogenic functional profile from *Eutypa dieback* one. A recent metabolomics experiment also showed indirectly that inoculation of specific endophytes strains causes a shift in grapevine secondary metabolism, and activation of defense pathways. Furthermore, it was confirmed the existence of strain-specific colonization patterns (Lòpez-Fernàndez et al., 2016).

Transcriptomic analysis of grapevine leaves and wood tissues revealed differentially expressed genes linked to latent *Neofusicoccum parvum* grapevine infection (Czemmel et al., 2015). However, this information is still largely incomplete.

In accordance with Busby et al. (2017), research should have different objectives to enable a reasoned, conscious and effective microbial exploitation, taking into account the impact of plant protection products on the quality of production and human health. In this direction, computational tools could be exploited to detect interactions among genes, pathogens and treatments, leading to a greater insight on their possible use and effects. Recent review on such methods is available in Lotfi Shahreza et al. (2017).

## 4. A case study: metabarcoding analysis of grapevine in chile

In order to show the power of bioinformatics metagenomic analysis in the study of grapevine, we developed a case study from 16S rRNA sequencing data from vineyards and adjacent forest areas in the Chilean territory (SRA Project: SRP110820; Miura et al. 2017). The study analyzed 6 vines from three Chilean geographic areas, and sclerophyllous trees from the adjacent forest area. Vines were sampled from both leaves and fruits. All analysis were replicated 3 times, leading to 54 samples, of which 36 grapevine and 18 controls. 2 samples were discarded due to quality issues. All analysis were conducted using QIIME 2 release 2017.9.

Paired-end Raw Illumina fastq files downloaded from SRA (SRP110820) were demultiplexed, quality filtered, and analyzed using QIIME. Reads containing one or more ambiguous base calls were discarded and truncated to a length of 200 nt. A subsequent filtering phase was performed using Deblur (Amir et al., 2017) to obtain putative error-free sequences from the original data. A phylogenetic tree was therefore built by making use of MAFFT (Katoh and Standley, 2013) and FastTree 2 (Price et al., 2010) allowing computation of subsequent diversity metrics.

**Figure 1:**
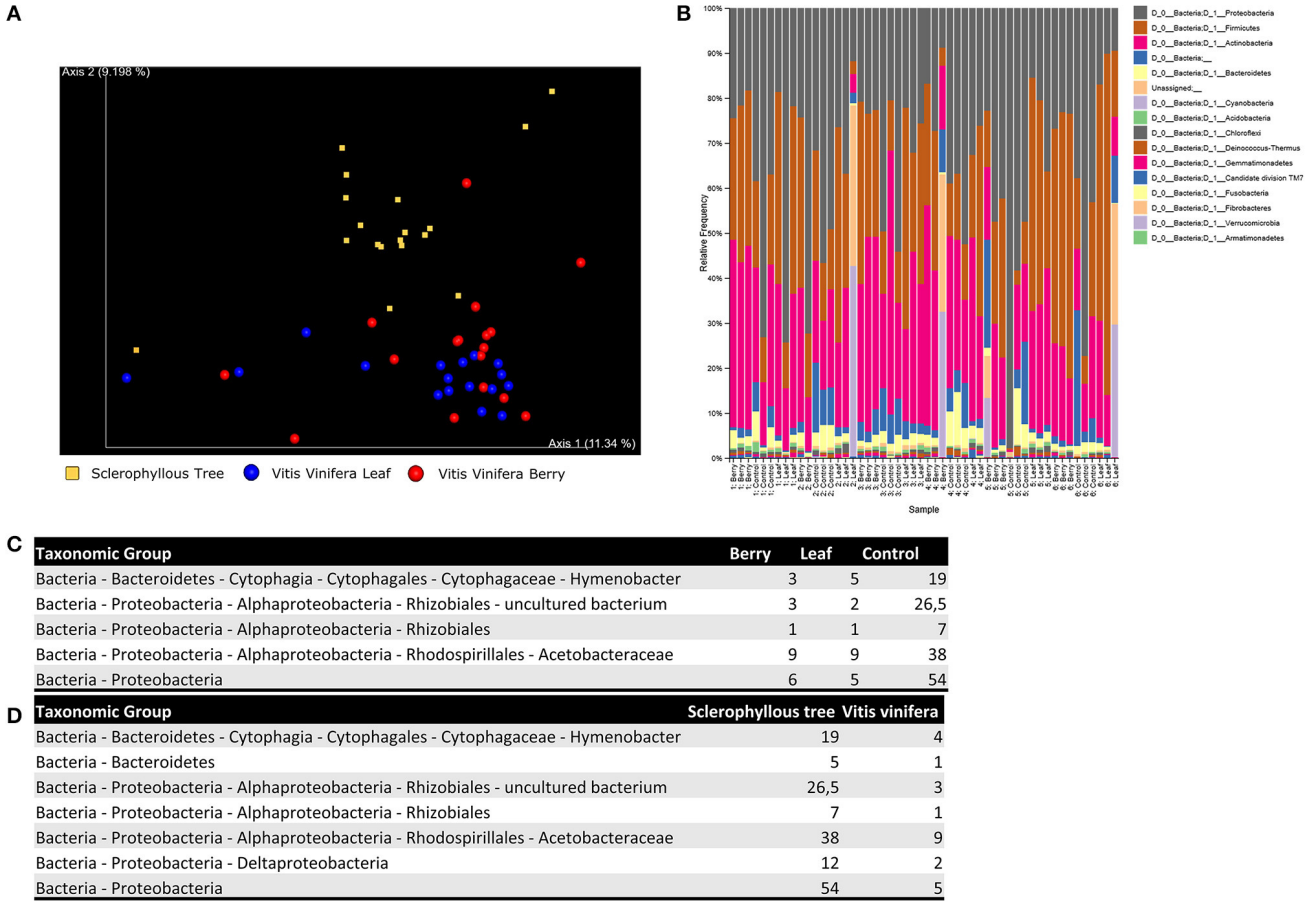
Results of the metabarcoding analysis of grapevine in Chile. The study analyzes 6 vines from three Chilean geographic areas, and sclerophyllous trees from the adjacent forest area. Vines are sampled from both leaves and fruits. All analysis are replicated 3 times, leading to 54 samples, of which 36 grapevine and 18 controls. 2 samples are discarded due to quality issues. Principal coordinates analysis plot is reported to visualize relationships between samples **(A)**. We also show the relative abundance of operational taxonomic units, assigned using QIIME feature classifier, at phylum level **(B)**, together with the median abundance values obtained for the most significant species in the differential analysis between sampling sites **(C)** and plant species **(D)**.

Alpha-diversity (within-sample species richness) and beta-diversity (between-sample community dissimilarity) estimates were calculated within QIIME using weighted UniFrac (Lozupone and Knight, 2005) distance between samples for bacterial 16S rRNA reads (evenly sampled at 1,000 reads per sample). Principal coordinates were computed from the resulting distance matrices to compress dimensionality intro 2D principal coordinate analysis (PCoA) plots, enabling visualization of sample relationships (Figure 1A). To determine whether sample classifications (host, sample site) contained differences in phylogenetic or species diversity, permutational MANOVA with 1,000 permutations was used to test significant differences between sample groups based on weighted UniFrac. No significant differences could be found in terms of alpha-diversity between hosts (*p* = 0.18) and sample sites (*p* = 0.41), and beta-diversity between sample sites (*p* = 0.13), however a significant difference in beta-diversity could be observed between host species (*p* = 0.03).

OTUs were assigned using QIIME feature classifier, which employs a Naive Bayes classifier to map each sequence to a taxonomy. The classifier was trained on a qiime-compatible Silva database (release 119), which includes sequences from 16S/18S rRNA. Any OTU representing less than 0.001% of the total filtered sequences was removed to avoid inclusion of erroneous reads, leading to inflated estimates of diversity. In Figure 1B we report the relative frequency of OTUs for each sample, sorted by site and host. Significant taxonomic differences between sample conditions were tested using ANCOM (Mandal et al., 2015). All results are available in Figures 1C,D.

Results shown in Figure 1B are consistent with Miura et al. We are able to retrieve the three main bacterial phyla (Actinobacteria, Firmicutes and Proteobacteria), and relative abundances are consistent. Furthermore, grapevine-related bacterial communities are similar to the phyllosphere of sclerophyllous trees when OTUs clustering is carried out at high bacterial taxonomic levels. This is not surprising since plants are known to show similar pattern at phylum level (Turner et al., 2013). Nonetheless, several differences can be detected at the genus, species or strain level. It is possible to make distinction between bacterial communities living on different plant species. This reflects the finely tuned metabolic adaptations required to live in symbiosis with the host (Figure 1D), but also between microbial communities living on different organs, reflecting the adaptations required to live in environment characterized by certain microclimatic conditions (Figure 1C).

## 5. Conclusions and perspectives

Gaining wider knowledge about plant-microbiota interactions at a molecular scale is an urgent task, since it can lead to the development of new biotechnological approaches, enhancing agriculture productivity and sustainability. NGS technologies together with bioinformatics are fundamental tools in this process. They have the potential to reveal new details concerning interactions between microbial communities and plants with unprecedented resolution. The characterization of microbial communities in grapevines at various conditions using high throughput sequencing technologies will sooner lead to the identification of disease-specific, cultivar-specific and climate-specific grapevine microbiota. Their exploitation in the field of viticulture could lead to the discovery of new applicable microbial strains, and could help us gaining a holistic and more complete view of plant-microbiome interactions at a genetic level. This would allow not only to potentiate antagonisms against phytopathogens and crop yield, but also to positively affect economically important traits, such as flowering time, and quality and flavor of grapes, must and wine.

## Author contributions

AP conceived developed and coordinated the research. AP, SA, AF, and RG analyzed the data. SA and GM wrote the paper. All authors read and approved the final version of the manuscript.

### Conflict of interest statement

The authors declare that the research was conducted in the absence of any commercial or financial relationships that could be construed as a potential conflict of interest.
